# Early intervention in autism spectrum disorder: The need for an international approach

**DOI:** 10.1111/dmcn.15327

**Published:** 2022-06-23

**Authors:** Amina Abubakar, Patricia Kipkemoi

**Affiliations:** 1 Institute for Human Development, Aga Khan University, Nairobi, Kenya; 2 INeurosciences Unit, KEMRI-Wellcome Trust Research Programme, Kilifi, Kenya

Globally, autism spectrum disorder (ASD) affects approximately 1 in 100 children.^1^ Ideally, a diagnosis is made with the onset of symptoms before 3 years of age; however, a diagnosis may sometimes be delayed until 6 years or later. There has been an increase in ASD research over the last few decades, with many systematic reviews and meta-analyses synthesizing research evidence for ASD interventions in children. Evidence tends to support the notion that intervention for ASD must occur as early as possible, close to the critical periods when early social and communication skills are developing. Therefore, early screening and intervention could improve the treatment outcomes of individuals with autism, not only helping them survive but to thrive.^2^

Franz et al.^3^ have conducted an overview of reviews to synthesize early intervention literature for very young children at risk for ASD with the aim of identifying which interventions have the strongest evidence base for impact. Researchers, including Franz et al., acknowledge that while many interventions impact child development, heterogeneity in child outcome measures (behavioural coding and structured observation assessments), treatment and intervention approaches, comparison groups, and participant profiles limit the extent to which we can concretely evaluate the effectiveness of interventions. Robust high-quality evidence is urgently needed to design international programmes that can address the sometimes complex and varied needs of individuals with autism.

Moreover, there are also inadequacies regarding the regional coverage of studies on ASD. The primary studies in these reviews were all from high-income settings (e.g. the USA and UK). Not a single primary study in the review by Franz et al.^3^ was identified from low- and middle-income countries (LMICs). There is an urgent need to generate evidence on the most appropriate intervention approaches for ASD in LMICs. Even as we advocate for more research on early intervention for children with ASD in LMICs, there is a need for further testing of interventions that provide high impact at a relatively low cost, given the limited resources in these settings and many competing priorities in overstretched health and educational settings. It has been argued that interventions that are community-based, empoweri caregivers and can be used to address the needs of children with different developmental delays may be the best options in these LMICs. In recent years the World Health Organization and Autism Speaks have led in the development and piloting of the Caregiver Skills Training programme (an intervention that addresses communication and behavioural problems across developmental conditions), which has the potential to address this gap and be used at scale in resource-constrained settings.^4^

In addition to the inclusion of applicable interventions in LMIC settings, there is ongoing discussion on the balance of benefits and harms of interventions in ASD research, with the consensus being that there is a dearth in the reporting of adverse events or observed harms.^5^ There is a need to address this issue and the high risk of bias through the application of fundamental standards in ASD intervention research.

In conclusion, Franz et al.^3^ have carried out a significant piece of work in early ASD intervention research by providing an overview of systematic reviews and primary studies therein. The authors call for a balance of research strategies to bridge the community implementation gap in early ASD intervention as there is a global disparity in who participates and benefits from intervention research.

## Data Availability

Not required.
